# The Electron Shuttle Critical Distance of Low Molecular Weight Organic Matters Accelerating Microbial Ferrihydrite Reduction

**DOI:** 10.3390/molecules30234559

**Published:** 2025-11-26

**Authors:** Qun Xue, Jingtao Duan, Zhen Yang, Guoxin Sun, Jie Jiang

**Affiliations:** 1 College of Environmental Science and Engineering, Beijing Forestry University, Beijing 100083, China; xq1013942152@163.com (Q.X.); jingtaoduan@u.nus.edu (J.D.); 2 Soil Environmental Science and Technology, Research Center for Eco Environmental Sciences, Chinese Academy of Sciences, Beijing 100085, China; zhenyang@rcees.ac.cn (Z.Y.); gxsun@rcees.ac.cn (G.S.); 3 State Key Laboratory of Regional and Urban Ecology, Research Center for Eco Environmental Sciences, Chinese Academy of Sciences, Beijing 100085, China; 4 College of Resources and Environment, University of Chinese Academy of Sciences, Beijing 101408, China

**Keywords:** natural organic matter, low molecular weight fraction, redox, electron shuttle critical distance

## Abstract

The redox activity of natural organic matter (NOM) is crucial for contaminants transformation in soils. Soil micropores (<2.5 nm) have limited accessibility for microorganisms and large NOM molecules; therefore, insoluble organic pollutants and heavy metals trapped in these micropores are usually reached by low molecular weight fractions (LMWF) of NOM. However, the mechanism of spatial electron transfer via electron shuttle of LMWF remains unclear. In this study, we separated low molecular weight fractions (LMWF < 3500 Da and LMWF < 14,000 Da) of Leonardite humic acids (LHA) and measured its acceleration of microbial ferrihydrite reduction. The results show that LMWF, as an electron shuttle, significantly accelerates the reduction in Fe (III), among which 3500-LMWF is the main fraction contributing to the acceleration. Additionally, 3D-EEM shows that quinone content was positively correlated with reduction efficiency, supporting its role as the key functional group. Based on the accelerating experiments, we determined an electron shuttling critical distance of 117.2 nm for LMWF LHA. These findings establish LMWFs as effective natural electron shuttles, providing a theoretical basis for understanding pollutant dynamics in soil micropores.

## 1. Introduction

Natural organic matter (NOM), such as humic substances (HS), is redox-active and can participate in many chemical reactions in soils [1]. Studies have shown that NOM can accept electrons from anaerobic microbial oxidation of inorganic or organic compounds such as hydrogen, organic acids, chlorinated or aromatic compounds [2,3,4], thereby affecting the reduction in Fe (III) and other metals and migration of organic pollutants [5]. Those reactions promote changes in the valence and solubility of contaminants in the soil by initiating or accelerating reduction and dissolution reactions [6,7,8,9]. Depending on the distribution of redox-active functional groups and the range of redox potentials [10,11,12], NOM can react with a bulk of pollutants in terrestrial and aquatic systems. Those processes are also affected by environmental (redox) conditions [8,10,13,14,15].

As most of the reactions in the soil occur on the surface of particles, the migration and transformation of contaminants, especially which trapped in soil pores, are closely related to the complexity and porosity of the soil. The accessibility of the pollutants filling of micropores depends mainly on the pore structure and shape, the size of the contaminant molecules and their interactions with the organic matter and with each other [16]. The International Association of Purification and Applied Chemistry (IUPAC) divided the soil pores into macropores (>50 nm), mesopores (2–50 nm), and micropores (<2 nm) [17,18]. Many pollutants have molecular weights between several thousand and tens of thousands of daltons and can enter micropores of soils less than 2 nm in diameter [17,19]. Therefore, there is a great risk of migration with groundwater flow and accumulation in soil micropores and will be hidden to prevent the detoxication of soil. It is particularly crucial to explore the mechanisms for migration and transformation of soil micropores pollutants.

HSs are supramolecular associations of self-assembled, relatively small heterogeneous molecules linked through hydrogen bridges and hydrophobic bonds [20,21,22]. The low molecular weight fractions (LMWF) of fulvic acids and humic acid could be isolated by membrane dialysis. It is confirmed that LMWF accounts for a very low proportion of HA, while per gram of carbon reducing capacities (RC) of reduced LMWF could be up to 33 times greater than that of the reduced bulk and retentate HA of dialysis [23]. LMWFs with greater RC are able to access micropores, where large HS molecules and microorganisms hardly exist, to react with contaminants there.

HS contains a large number of functional groups, among which quinone is considered to be the most important redox functional group [24]. The characterization of these functional groups has been carried out by three-dimensional excitation-emission (3D-EEM) spectroscopy [25,26,27]. The change in fluorescence intensity with both excitation and emission wavelength can be obtained to fully describe HS complex multi-component system [21,28]. Specific fluorescence components have been reported to be the characteristics of quinone [29].

Jiang and Kappler demonstrated that electron shutting is only possible at concentrations of dissolved HS of at least 5–10 mg C/L [7]. Since studies have shown that LMWFs have strong RC [23], whether they, with certain concentrations, can stimulate the process of microbial-metal ions reduction as electron shuttle and what the mechanism of spatial electron transfer is has become the focus of our attention. This could be a significant explanation for the migration and transformation of contaminants in soil micropores.

In this study, we obtained 3500 Da and 14,000 Da LMWF molecules and characterized their molecular structure using 3D-EEM spectroscopy. Accelerated ratios of microbial ferrihydrite reduction rate in the presence of 3500-LMWF and 14,000-LMWF LHA were calculated. The critical distance of electron shuttle by LMWF LHA was subsequently confirmed based on the establishment of HA electron transfer space model. It serves as a reference to provide us with a scientific basis for intervening in and regulating the environmental processes of soil micropores.

## 2. Results

### 2.1. Dialysis Process of the LMWF LHA

To simulate releasing processes of HS LMWF through a soil micropore environment, dialysis experiments were conducted to separate the different molecular weight fractions from bulk LHA. The TOC contents for the LWMF LHA that were able to pass through micropores with a diameter of 1.25 or 2.5 nm (e.g., MW < 3500 Da; MW < 14,000 Da) are shown in Table 1.

The TOC contents of 3500-LMWF LHA and 14,000-LMWF LHA increased gradually from 3.137 to 6.738 mg C/L and 7.128 to 12.145 mg C/L, respectively. Additionally, 14,000-LMWF LHA possesses more organic carbon due to molecules between 3500 and 14,000. Moreover, TOC content differences between the LWMF LHA and bulk LHA were shown in Table 1. LMWF content accounts for only 1.5–5.5% of the TOC content in the bulk LHA molecules.

### 2.2. LMWF Accelerating Microbial Ferrihydrite Reduction

Previous study has demonstrated that LMWF RCs per gram of carbon were up to 33 times greater than either those of the bulk HA or the retentate [23]. In order to explore whether LMWF can work as electron shuttle between the *Shewanella oneidensis* MR-1 and ferrihydrite, especially at low concentrations, we carried out microbial ferrihydrite reduction experiments to figure out electron shutting of LMWF LHA with different molecular weights and molecular size.

**Figure 1:**
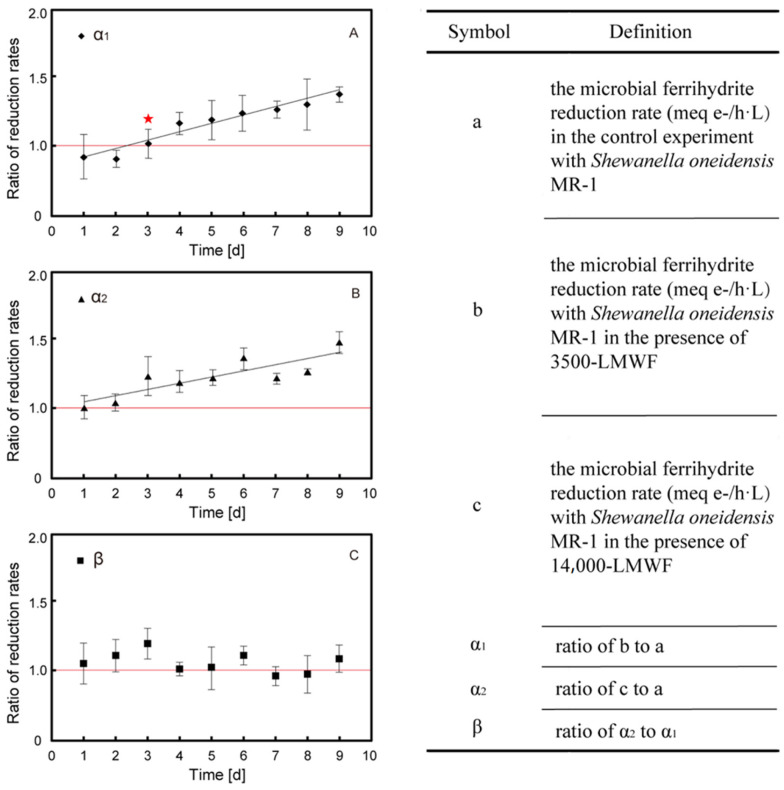
(**A**) the ratio of microbial ferrihydrite reduction rate (◇) in the presence of 3500-LMWF LHA to it in the absence of HS (star point means start accelerating). (**B**) the ratio of microbial ferrihydrite reduction rate (△) in the presence of 14,000-LMWF LHA to it in the absence of HS. (**C**) the microbial ferrihydrite reduction rate (□) with 14,000-LMWF LHA to that with 3500-LMWF LHA. The error bars represent the standard deviation of three independent reactors.

Both α1 and α2 are identified as Fe (III) reduction rate increased coefficient in the presence of LMWF LHA. Figure 1A,B show that α1 and α2 continuously increased with the increase in dialysis time as follows: α1 was range of 0.96–1.35 and α2 was range of 1.01–1.44 (Table 2). This result indicated that LMWF LHA (α_1_ and α_2_ > 1) can accelerate microbial ferrihydrite reduction compared to control experiments. Furthermore, 3500-LMWF LHA did not accelerate the reduction until 3-day dialysis, whose TOC > 2.3 mg C/L. This acceleration of reduction is induced by electron shuttle, and the threshold of TOC content being able to accelerate microbial ferrihydrite reduction is lower than 5 mg C/L reported in previous study about bulk HA [7]. In order to investigate the effect of molecular size on the reduction rates, ratio of α_2_ and α_1_ was calculated as β (Figure 1C). The β value fluctuated around one independence of dialysis time. It is indicated that the LMWF with molecular weight between 3500 and 14,000 Da could barely stimulate the reduction, and 3500-LMWF LHA is the main contributor to accelerated microbial reduction in ferrihydrite.

### 2.3. Fluorescence Characteristics of 3500- and 14,000-LMWF Samples

**Figure 2:**
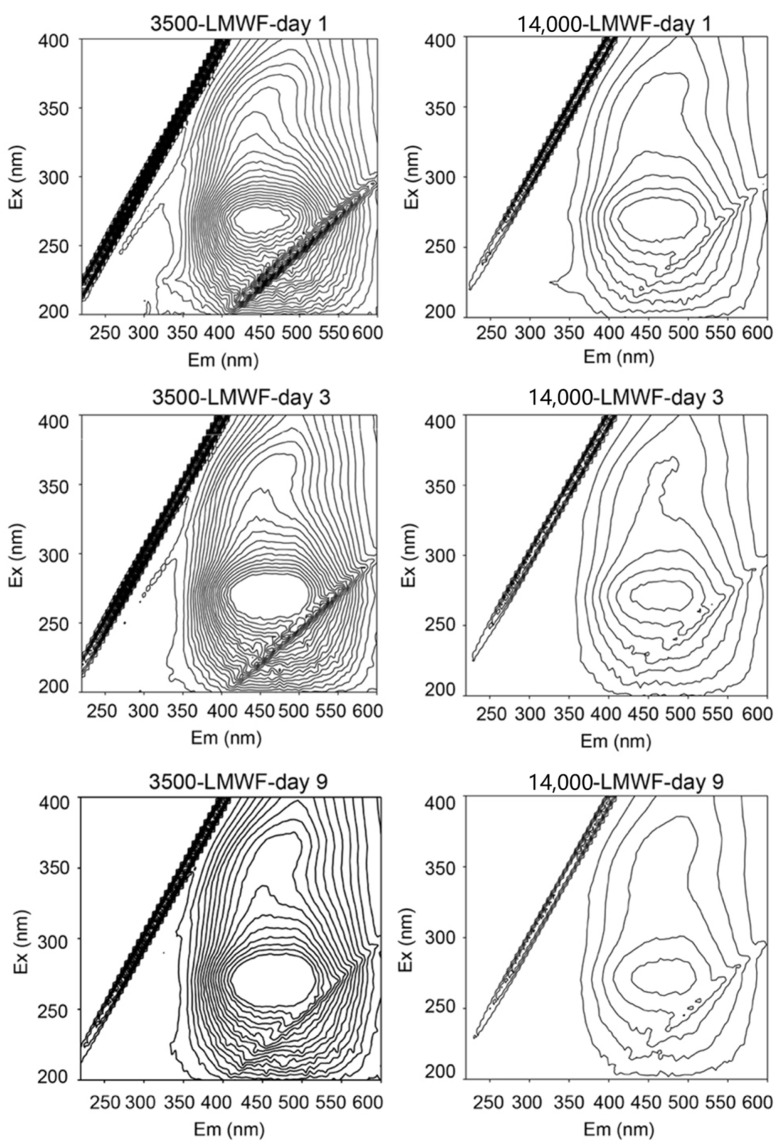
The 3D-EEM spectra of 3500- and 14,000-LMWF samples during dialysis (day 1, 3, and 9).

Following the comparison of the effects of these two LMWFs on microbial ferrihydrite reduction, we employed fluorescence spectroscopy to identify the structural features responsible for the observed differences. Specifically, we investigated how the structures of these two LMWFs differed after varying periods of dialysis under identical TOC conditions. The fluorophores of LMWF in Figure 2 include protein-like fluorophores including tryptophan-like peak (Ex: 270–280 or <240 nm; Em: 330–368 nm) and a tyrosine-like peak (Ex: 270–275 nm; Em: 304–312 nm) and humic-like fluorophores (Em > 400 nm). Specifically, humic-like fluorophores are related to quinone groups. When the wavelength is greater than 400 nm, Ex wavelength at 250–300 nm and 330–400 is caused by quinonoid π-π* transition. For all of LMWF samples, major quinone-like fluorophores and minor protein-like fluorophores were detected. Higher relative fluorescence intensities were detected in quinone-like fluorophores (1.13–1.96 a.u., Table 3) than in protein-like fluorophore (0.08–0.24 a.u., Table 3), suggesting that quinone-like fluorophore is the predominant fluorophore in LMWF samples.

During dialysis from day 1 to 9, a slight redshift of 5–10 nm occurred at the Ex/Em position of 3500-LMWF LHA, and the Em of 14,000-LMWF LHA showed a significant redshift of 25 nm. These results suggested that large levels of conjugated chromophores in quinone-like groups emerged. Additionally, an increase in the number of quinone fluorophores can shorten the electron transfer distance between adjacent functional groups, thereby accelerating electron shuttling. The relative fluorescence intensities of quinone-like fluorophores in 14,000-LMWF were lower than that in 3500-LMWF. This implies that, under the same TOC, 14,000-LMWF had less quinone-like fluorophore than 3500-LMWF. This also indicates that LMWF with less than 3500 Da is the main redox active component in 14,000 Da LMWF, which is consistent with the results of the experiment in Section 2.2. These results suggested that LMWF LHA accelerates the reduction in ferrihydrite by electron shuttling between cells and ferrihydrite, and the quinone-like fluorophores of LMWF LHA are related to the reduction rate.

### 2.4. The Critical Distance of Electron Shuttle in LMWF HA Molecules

**Figure 3:**
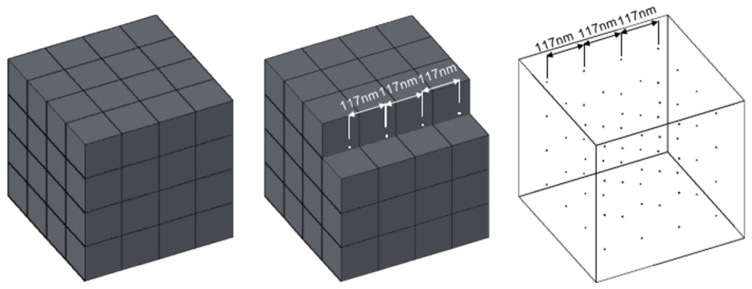
Uniform distribution model of LMWF unit volume.

According to results of acceleration experiments, we found that when TOC was 2.305 mg C/L of 3500-LMWF in the reactor, LMWF LHA started to have a stimulation effect (α_1_ > 1, Figure 1A). This result indicated that there is a limit distance between adjacent molecules of LMWF LHA molecules that impacts electron shuttle via LMWF LHA molecules and thus influences rates of microbial ferrihydrite reduction. We believe that this condition in the reactor is the critical condition for the electron shuttle, so we can evaluate the critical distance between adjacent LMWF LHA molecules. The reactor maintains shaking during the reaction process to ensure the uniform distribution of LMWF LHA within the reactor; therefore, a spatial distribution model of LMWF LHA molecules can be developed. An occupied volume of a single LMWF LHA molecule was calculated based on the number of LMWF LHA in the unit volume (1L) cube, and the distances between adjacent HA molecules can be obtained (Figure 3).

First, according to ^12^C atomic mass is 1.993 × 10^−26^ kg and the definition of Dalton, we can calculate the mass of 3500-LMWF:

1 Da = 1/12 × ^12^C = 1/12 × 1.993 × 10^−26^ = 1.661 × 10^−21^ mg, (1)

3500 Da = 3500 × 1.661 × 10^−21^ = 5.814 × 10^−18^ mg, (2)

In the LHA composition, C accounts for 63.81%, so the mass of C in 3500-LMWF is 3.709 × 10^−18^ mg. In this reactor, the TOC was 2.305 mg C/L, the total carbon mass of LMWF in the 1 L reactor is 2.305 mg C. We use 3500 Da as the standard molecular weight of the fractions; then, we can calculate number (*N*) of 3500-LMWF LHA molecules in per unit volume:

*N* = 2.305/(3.709 ×10^−18^) = 6.215 × 10^17^, (3)

Occupied volume (V) of each HA molecules in the 1L reactor can be calculated:

V = 1/(6.215 ×10^17^) = 1.610 × 10^−18^ dm^3^, (4)

Center distance (CD) between adjacent 3500-LMWF LHA molecules can be obtained:

CD = (V) ^1/3^ = (1.610 ×10^−18^) ^1/3^ = 1.172 ×10^−6^ dm = 117.2 nm. (5)

Through above calculation, the critical distance of 3500-LMWF LHA electron shuttle is 117.2 nm. That is, when the distance between two molecules is less than 117.2 nm, it can act as an electron shuttle to accelerate the microbial ferrihydrite reduction reaction.

## 3. Discussion

Small-molecule organic substances in the environment (such as quinones, flavins, and humus fragments) can act as electron shuttles to transfer electrons between microorganisms or between microorganisms and minerals. The efficiency of this process directly depends on the shuttling efficiency of electrons between molecules. In this study, we successfully separated different molecular weight fractions which could flow into and out of the soil micropores to react with contaminants hidden in it. The LMWF LHA stimulation effect suggests that it has a high potential to contribute to pollutant reduction at soil micropores. The in-depth study of the redox active molecular structure of LMWF HA and its electron shuttle mechanism is of great significance for understanding the process of transformation or degradation of organic pollutants and heavy metals in soil micropores.

At low concentrations, even if a portion of HA is reduced, due to its small quantity, the probability of collision with minerals is low, and an efficient electron shuttle cannot be formed. Only when the concentration reaches the critical point and there is a sufficient number of free molecules in the solution, with the average distance between them reduced to be comparable to the effective electron shuttle distance, do the reduced HA molecules not need to diffuse over long distances. It ensures that the HA reduced by microorganisms can continuously and efficiently transport electrons to the mineral surface through contact reaction or electron hopping with adjacent oxidized HA molecules [30].

The quinone groups on HA are the main electron-receiving functional groups. The content of redox active groups in HA from different sources varies, resulting in different electron shuttling capabilities and thus affecting their relative efficiency as electron shuttles. The quinone group information obtained by 3D-EEM characterization in this study is positively correlated with the effect of LMWF LHA in accelerating the microbial reduction in ferrihydrite, which also confirms the above statement.

Based on the experimental results, we calculated that the critical distance of the electron shuttle of 3500-LMWF LHA is 117.2 nm. This value provides a crucial quantitative benchmark for understanding the effective range of redox-active low-molecular-weight organic matter. Although natural organic matters have inherent heterogeneity in composition and structure, the critical distance values of organic matters from different sources may fluctuate within a certain range. However, the specific value obtained in this study establishes a quantifiable benchmark, revealing the spatial-scale characteristics of such substances in achieving effective electron transfer at the microscopic scale.

## 4. Materials and Methods

Source and preparation of HA solution: Leonardite humic acids Standard (LHA) was purchased from the International Humic Substances Society (IHSS). Dissolve an appropriate amount of LHA with a sterilized phosphate-buffer solution (PP buffer; pH7, 50 mM) to ensure a concentration of 0.5 mg/mL. In order to avoid photochemical action, LHA solution should be stored in the refrigerator at 4 °C in the dark [7].

Dialysis experiment: According to the soil pore size, MD34-3500 (pore size 1.25 nm) and MD34-14,000 (pore size 2.5 nm) dialysis bags (VISKASE, Danbury, CT, USA) were selected. Completely immerse the dialysis bag containing 50 mL of 0.5 mg/mL bulk HA in sterilized 200 mL of PP buffer solution and stir in the dark at 500 rpm. LMWF and retentates were collected at dialysis time 24 h, 48 h, 72 h up to 216 h. All samples stored at 4 °C in the dark. Bulk, 3500-LMWF, 14,000-LMWF, 3500-retentate, 14,000-retentate refer to LHA solutions having low molecular weight fractions less than3500 Da, less than 14,000 Da, and the macromolecular polymer remaining in the dialysis bag.

TOC determination: The total organic carbon (TOC) contents of the bulk, 3500-LMWF, 14,000-LMWF, 3500-retentate, 14,000-retentate LHA were determined by TOC-V CSN analyzer (SHIMADZU, Kyoto, Japan) with detection limit of 50 μg/L.

Ferrihydrite solution: Ferrihydrite solution was prepared by dissolving 2.5 g of Fe(NO_3_)_3_·9H_2_O in 25 mL of deionized water with continuous stirring. The pH was initially adjusted to 2.5–3.0 using approximately 15 mL of 1 mol/L KOH. It was then titrated to a final pH of 7.3 using 1 mol/L KOH. The solution was transferred to a centrifuge tube and subjected to four cycles of centrifugation at 4000 rpm for 30 min, each time discarding the supernatant. The resulting precipitate was washed with distilled water to collect the brown ferrihydrite suspension.

*Shewanella* Basal Medium (SBM): SBM was prepared by dissolving the following components in approximately 800 mL of deionized water: 0.295 g of K_2_HPO_4_·3H_2_O, 0.225 g of KH_2_PO_4_, 0.46 g of NaCl, 0.225 g of (NH_4_)_2_SO_4_, 0.057 g of MgSO_4_, 2.38 g of HEPES, and 3.73 mL of 60% sodium lactate. The mixture was stirred until all components were completely dissolved. The volume was then adjusted to 1 L with deionized water. The pH was adjusted to 7.2 using 1 mol/L KOH, and the medium was sterilized by autoclaving at 121 °C for 30 min.

Determination of microbial ferrihydrite reduction: The study was carried out under anaerobic conditions with a pH value of 7. Ferrihydrite was prepared according to Cornell and Schwertmann [31]. The effect of different molecular weights HA on the iron reduction process of *Shewanella oneidensis* strain MR-1 (OD = 1) was studied by adding different molecular weight humic components to a reactor comprising SBM [32], strains, and ferrihydrite (1 mM, 30 °C, 220 rpm). These HA fractions were fully dissolved and homogeneously distributed in the reaction systems. Samples were taken per hour and immediately mixed with 1 M hydrochloric acid (HCl) (up to 3 h). The amount of reduced iron [Fe (II)] was quantified spectrophotometrically (ferrozine assay [7,33]).

Three-dimensional excitation-emission spectroscopy: A sample of HA of different molecular weights was taken into a four-pass cuvette (inner diameter 10 mm), and the three-dimensional fluorescence characteristics of HA were performed using a molecular fluorescence analyzer (F7000) equipped with a 1500 W Xe lamp (HITACHI, Tokyo, Japan). Spectrometer parameters were set to Excitation Wavelength (Ex) range of 200–400 nm, Emission Wavelength (Em) range of 220–600 nm, and scan speed set to 1200 nm/min. The excitation wave slit width of bulk and retentate component was set to 1.0 nm, and the emission wave slit width was set to 5.0 nm. The excitation and the emission wave slit width of LMWF were all set to 1.0 nm.

## Figures and Tables

**Table 1:** TOC of bulk LHA and percentage of two different molecular weight fractions (in % of the bulk LHA TOC).

Time [Day]	3500-LMWF TOC [mg C/L]	3500-LMWF/Bulk [%]	14,000-LMWF TOC [mg C/L]	14,000-LMWF/Bulk [%]
1	3.14	1.44	7.13	3.27
2	4.73	2.17	7.79	3.57
3	4.61	2.11	9.14	4.19
4	5.28	2.42	10.27	4.71
5	5.77	2.65	10.00	4.59
6	6.48	2.97	11.36	5.21
7	6.45	2.96	11.07	5.08
8	6.65	3.05	11.81	5.42
9	6.74	3.09	12.15	5.57

**Table 2:** Ratio of reduction rate of LMWF LHA participating in the microbial ferrihydrite reduction.

3500-LMWF TOC ^1^ [mg C/L]	Ratio	14,000-LMWF TOC ^1^ [mg C/L]	Ratio
1.570	0.96	3.565	1.01
2.365	0.95	3.895	1.04
2.305	1.04	4.570	1.21
2.640	1.17	5.135	1.18
2.885	1.19	5.000	1.21
3.240	1.23	5.680	1.34
3.225	1.25	5.535	1.21
3.325	1.29	5.905	1.25
3.370	1.35	6.075	1.44

^1^ TOC refers to the concentrations of LMWF LHA in the reactors.

**Table 3:** Characteristic of fluorophores with 3500- and 14,000-LMWF LHA samples during dialysis (day 1, 3, and 9).

HA Samples	Dialysis Time [d]	Ex/Em [nm]	Intensity ^1^ [a.u.]	Fluorophores	Peak Type
3500-LMWF	1	275/305	0.10	protein-like	tyrosine-like
		220/330	0.07	protein-like	tryptophan-like
		270/450	1.36	humic-like	quinone-like
	3	275/305	0.06	protein-like	tyrosine-like
		210/335	0.06	protein-like	tryptophan-like
		270/450	1.36	humic-like	quinone-like
		355/460	0.60	humic-like	quinone-like
	9	275/305	0.05	protein-like	tyrosine-like
		220/330	0.04	protein-like	tryptophan-like
		270/450	1.11	humic-like	quinone-like
		360/470	0.55	humic-like	quinone-like
14,000-LMWF	1	275/305	0.08	protein-like	tyrosine-like
		225/345	0.16	protein-like	tryptophan-like
		270/460	1.20	humic-like	quinone-like
		360/460	0.55	humic-like	quinone-like
	3	275/305	0.04	protein-like	tyrosine-like
		225/340	0.07	protein-like	tryptophan-like
		270/485	0.97	humic-like	quinone-like
		365/485	0.51	humic-like	quinone-like
	9	275/305	0.03	protein-like	tyrosine-like
		225/340	0.05	protein-like	tryptophan-like
		270/485	0.70	humic-like	quinone-like
		365/485	0.43	humic-like	quinone-like

^1^ The intensity refers to the relative fluorescence intensity. The intensities were normalized to the area under the emission at 355 nm determined in PP buffer.

## Data Availability

Dataset available on request from the authors.
